# Symptom Reporting Behaviors, Symptom Burden, and Quality of Life in Patients with Hormone Receptor–Positive Breast Cancer Undergoing Adjuvant Endocrine Therapy

**DOI:** 10.3390/curroncol32110599

**Published:** 2025-10-24

**Authors:** Ece Ulukal Karanci, Halil Göksel Güzel, Banu Öztürk

**Affiliations:** Department of Clinical Oncology, Antalya Education and Research Hospital, University of Health Sciences, 07100 Antalya, Turkey; hgguzell@gmail.com (H.G.G.); drbanutr@yahoo.com (B.Ö.)

**Keywords:** adjuvant endocrine therapy, hormone receptor–positive breast cancer, quality of life, symptom burden, patient-reported outcomes

## Abstract

**Simple Summary:**

Many women with hormone-sensitive breast cancer undergo long-term hormone therapy to prevent cancer recurrence. While this treatment saves lives, it can cause difficult side effects, such as joint pain, hot flashes, and mood changes, that significantly lower a patient’s quality of life. Patients often do not inform their doctors about these problems until they become severe. This study aimed to understand which patients experience the worst side effects and how their symptom-reporting behaviors relate to their well-being. We surveyed 191 women undergoing this therapy at a hospital in Antalya, Turkey. Our results showed that patients who had previously received chemotherapy or were taking medication to suppress ovarian function had a much higher burden of symptoms. We also found that patients who reported their side effects to their doctor, used alternative medicines, or received psychological support tended to have more severe symptoms and lower quality of life. This suggests that patients who suffer the most actively seek help. This study highlights the importance of proactively asking patients about their side effects rather than waiting for them to report them. By regularly monitoring symptoms, healthcare teams can offer better and more personalized support to improve the daily lives of breast cancer survivors.

**Abstract:**

**Background**: Adjuvant endocrine therapy (AET) enhances survival outcomes in hormone receptor–positive (HR+) breast cancer. However, this treatment is associated with toxicities that may adversely affect the quality of life (QoL) and impact patient–physician communication. A thorough understanding of symptom-reporting behaviors is essential for optimizing survivorship care. **Methods**: This cross-sectional study surveyed 191 female patients with HR+ breast cancer undergoing adjuvant AET (tamoxifen or aromatase inhibitors ± ovarian function suppression [OFS]) at Antalya Training and Research Hospital between July and August 2025. QoL, symptom burden, and adverse event (AE) reporting behaviors were assessed using validated instruments (European Organization for Research and Treatment of Cancer Quality of Life Questionnaire C30 [EORTC QLQ-C30], adapted Patient-Reported Outcomes version of the Common Terminology Criteria for Adverse Events [PRO-CTCAE]). Categorical variables were compared using chi-square tests, and multivariate analyses were performed using logistic regression. **Results**: The median age was 54 years (interquartile range [IQR]: 46–61 years). The following independent variables were identified as predictors of a higher symptom burden: prior chemotherapy (odds ratio [OR]: 3.75; 95% confidence interval [CI]: 1.46–9.69; *p* = 0.006), OFS use (OR: 3.29; 95% CI: 1.51–7.15; *p* = 0.003), AE reporting to physicians (OR: 3.52; 95% CI: 1.80–6.88; *p* < 0.001), and complementary and alternative medicine (CAM) use (OR: 7.27; 95% CI: 1.57–33.63; *p* = 0.011). Independent predictors of poor QoL included receiving psychological support (OR: 0.36; 95% CI: 0.19–0.67; *p* = 0.002) and AE reporting (OR: 0.28; 95% CI: 0.13–0.64; *p* = 0.001). **Conclusions**: Symptom burden and QoL in patients with HR+ breast cancer receiving AET are influenced by clinical history, including chemotherapy and OFS; behavioral factors, such as reporting behaviors; and supportive care, including CAM and psychological support. The routine integration of patient-reported outcomes and proactive symptom monitoring is crucial for delivering personalized and effective survivorship care.

## 1. Introduction

Breast cancer is the most prevalent malignancy among women globally, with hormone receptor-positive (HR+) subtypes constituting approximately 70% of all cases [1]. Adjuvant endocrine therapy (AET) plays a crucial role in significantly reducing the risk of recurrence and enhancing survival rates [2]. International guidelines incorporate combination regimens involving tamoxifen, aromatase inhibitors (AI), and ovarian function suppression (OFS) as standard treatment options [3].

Nevertheless, prolonged AET use is associated with toxicities that may adversely affect the quality of life, including vasomotor symptoms, musculoskeletal pain, gynecological issues, and psychosocial effects [4]. Although large, randomized trials (e.g., SOFT and TEXT) have validated the efficacy of various AET regimens, it has been demonstrated that those containing OFS, in particular, are associated with a heightened symptom burden, potentially compromising patient adherence [5]. Patient-reported outcomes (PROs) are essential for systematically and reliably capturing patients’ experiences of adverse effects [6]. However, in real-world clinical practice, a substantial proportion of patients do not spontaneously report their symptoms to clinicians; reporting is predominantly initiated when symptom severity escalates [7]. This situation underscores the necessity of a thorough examination of how physicians report symptoms to identify potential issues in patient treatment.

This study aimed to assess the frequency of hormone therapy-related side effects, their impact on quality of life (QoL), and specifically the behavior of reporting symptoms to physicians in adult women diagnosed with HR+ breast cancer and undergoing adjuvant hormone therapy (tamoxifen or aromatase inhibitor ± OFS). Our hypothesis posits that the reporting of side effects to physicians is correlated with symptom burden and QoL, and that certain clinical/behavioral factors [history of chemotherapy, use of Traditional and Alternative Therapies (TAT), and receipt of psychological support] play a decisive role in this relationship.

## 2. Materials and Methods

### 2.1. Study Population

This study included patients diagnosed with estrogen receptor-positive invasive breast cancer who were undergoing adjuvant hormonal therapy and were monitored at the Medical Oncology Outpatient Clinic of Antalya Training and Research Hospital. Questionnaires were administered between July and August 2025. The inclusion criteria were as follows: diagnosis of estrogen receptor-positive invasive breast cancer, age greater than 18 years, receipt of adjuvant hormonal therapy, completion of (neo) adjuvant cytotoxic chemotherapy and/or adjuvant radiotherapy at least three months prior, if applicable, availability of clinical and pathological data, and provision of both verbal and written consent to participate in the questionnaire study. The exclusion criteria were irregular follow-up, clinical, pathological, and/or radiological evidence of breast cancer recurrence, stage 4 disease at diagnosis, and refusal to provide consent for participation in the survey. A total of 192 patients met the inclusion criteria; however, one patient was excluded due to insufficient data and follow-up loss. Consequently, the final analyses were conducted on 191 patients (n = 191).

Study Surveys and Data Collection Patients were required to complete four distinct questionnaires addressing clinical and demographic information, quality of life, symptom burden associated with hormonal therapy side effects, perception of these side effects, and their reporting behavior to physicians. The questionnaires employed in this study were as follows: (1) A 14-item general information questionnaire capturing patients’ clinical and demographic data, including educational status, marital status, adherence to hormonal treatment, and receipt of psychological support. (2) A 5-item quality of life questionnaire designed to evaluate overall quality of life, utilizing a 5-point Likert-type scale for each item (not at all, a little, moderate, very, and extremely). (3) A 6-item questionnaire was used to identify symptoms resulting from the side effects of hormonal treatment, employing a 5-point Likert-type scale for each item (not at all, a little, moderate, very, and extremely). (4) To assess patients’ perceptions of side effects and their reporting behavior to physicians, a 6-item questionnaire with yes/no response options was utilized. In the development of Surveys 2, 3, and 4, items from the European Organization for Research and Treatment of Cancer Quality of Life Questionnaire C30 (EORTC-QLQ-C30), EORTC-QLQ-BR23, and Patient Reported Outcomes—Common Terminology Criteria for Adverse Effects (PRO-CTCAE) questionnaires were evaluated to formulate analogous items. Official online permission was obtained from the relevant organizations. Additional data utilized in the study, not included in the questionnaires, were obtained from patient follow-up files and the hospital electronic information management system.

A composite QoL score was derived from the responses to the QoL questionnaire (No. 2) included in the study, while a composite symptom burden score was generated from the responses to the symptom burden questionnaire (No. 3). The question “Do you have difficulty performing your daily tasks?” (Question 2) was identified as inconsistent with the other questions during the Cronbach’s alpha sensitivity analysis and was consequently excluded from the composite QoL score calculation. Breast cancer staging was conducted using the American Joint Committee on Cancer (AJCC) Staging System, 8th edition. All patients received treatment in accordance with the most appropriate method according to the current treatment guidelines. This study was conducted in accordance with the principles of the Universal Declaration of Human Rights. Ethical committee approval for this study was obtained on 3 July 2025, from the Scientific Research Ethics Committee of Antalya Training and Research Hospital (Approval No: 2025-216).

### 2.2. Statistical Analysis

The data collected in this study were analyzed using IBM SPSS Version 24.0. Continuous variables are presented as median values with interquartile ranges (IQR) (25th–75th percentile) in parentheses, and categorical variables are represented as percentage frequencies. For the calculation of the composite QoL and composite symptom scores, responses on the 5-point Likert scale in the questionnaire were assigned scores of 0, 25, 50, 75, and 100, indicating a range from low to high QoL and symptom scores. The internal consistency of the QoL and symptom burden questionnaires was assessed using Cronbach’s alpha analysis. Subsequently, the arithmetic mean of the responses provided by each participant was calculated. Scores were not calculated for participants who left three or more questions unanswered on each questionnaire. Nevertheless, no participants were excluded from the study because of incomplete questionnaires. The chi-square test was used to compare categorical variables in the univariate analysis of factors influencing low or high composite symptom burden and QoL scores. Variables with a chi-square *p*-value of less than 0.4 were included in the multivariate analysis. Multivariate analyses were conducted in two stages using the logistic regression method, with the dependent variables being low and high composite symptom burden scores and low or high composite quality of life scores. The odds ratio (OR) was calculated for each variable, with a 95% confidence interval (CI). Statistical significance was set at *p* < 0.05.

### 2.3. Findings

The study included 191 women diagnosed with estrogen receptor-positive breast cancer, all of whom received adjuvant hormonal therapy (n = 191). The median age of the participants was 54 years, with an IQR of 46–61 years. Among these patients, 73 (38.2%) were premenopausal or perimenopausal, and 118 (61.8%) were postmenopausal. All participants exhibited an Eastern Cooperative Oncology Group Performance Status (ECOG PS) score of 0 or 1. Of the total cohort, 71 patients (37.2%) were administered tamoxifen, and 120 patients (62.8%) received aromatase inhibitors. OFS was performed in 56 patients (29.3% of total patients) (Table 1).

**Figure 1:**
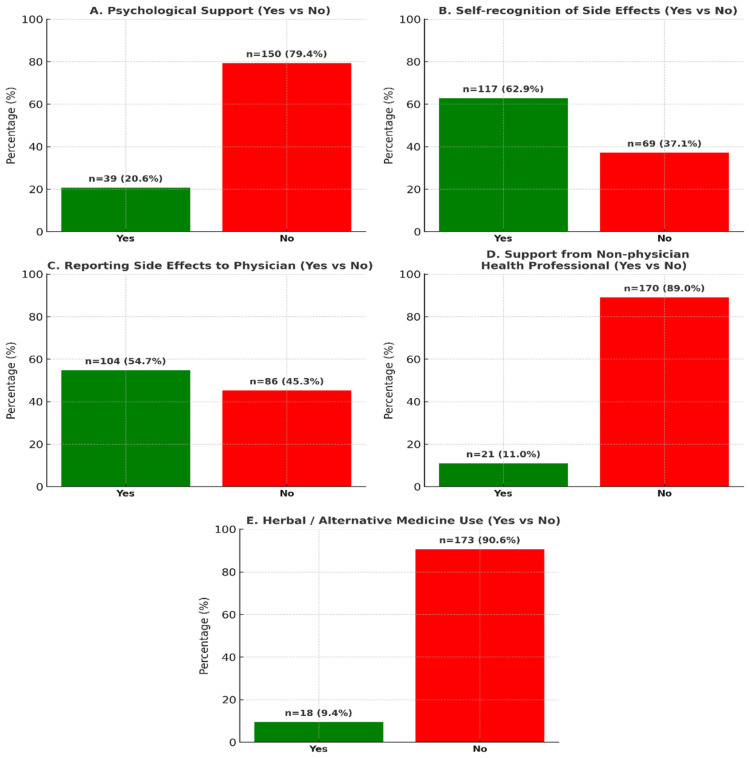
Patterns of Patient Behavior in Response to Side Effects of Hormone Therapy.

In this study, 117 participants (61.4%) reported awareness of the side effects associated with hormone therapy. Of these, 104 individuals (54.5%) communicated these side effects to their physicians, whereas 86 participants (45.0%) did not disclose this information to their healthcare providers. A subset of 22 patients (11.5%) expressed difficulty in articulating the side effects to their doctors, while 106 participants (55.5%) indicated that their physicians acknowledged the side effects and proposed solutions for them. Additionally, 27 patients (14.1%) contemplated discontinuing treatment due to side effects, and five individuals (2.6%) actually ceased treatment. Furthermore, 21 patients (11%) sought assistance from healthcare professionals other than their primary doctor, and 18 participants (9.4%) resorted to herbal or alternative medical products in response to side effects (Figure 1).

The internal consistency of the composite symptom burden score was evaluated using Cronbach’s alpha, yielding a value of α = 0.735, which indicates the acceptable reliability of the scale. The median value for the composite symptom burden score was 45.83 (IQR: 25–58.33).

Patients were categorized into two groups based on their scores: those with scores below the median were classified as having low symptom scores, while those with scores at or above the median were classified as having high symptom scores. Univariate analyses were conducted to identify predictors of low and high symptom scores, revealing significant associations with age (*p* = 0.001), menopausal status (*p* = 0.005), history of chemotherapy (*p* = 0.013), type of hormone therapy (*p* = 0.025), use of ovarian function suppression (OFS) (*p* = 0.002), receipt of psychological support (*p* = 0.022), reporting of side effects to a physician (*p* < 0.001), and the use of herbal products or alternative medicine methods (*p* = 0.007). In the multivariate analysis, age (*p* = 0.240), menopausal status (*p* = 0.759), type of hormone therapy (*p* = 0.290), and receipt of psychological support (*p* = 0.087) did not demonstrate independent predictive values for sexual dysfunction. Conversely, a history of chemotherapy was significantly associated with a higher symptom burden (OR: 3.75, 95% CI: 1.46–9.69, *p* = 0.006). Similarly, patients using OFS exhibited a significantly higher symptom burden (OR: 3.29, 95% CI: 1.51–7.15, *p* = 0.003). Reporting side effects to a physician was also associated with a higher symptom burden (OR: 3.52, 95% CI: 1.80–6.88, *p* < 0.001). Furthermore, patients who used herbal products or alternative medicine had a significantly higher composite symptom burden score than those who did not (OR: 7.27, 95% CI: 1.57–33.63, *p* = 0.011) (Table 2).

Composite QoL Score and Determinants The internal consistency of the composite symptom burden score was evaluated using Cronbach’s alpha coefficient, yielding a value of α = 0.717. The median score was 56.25 (43.75–68.75). Based on this median value, patients were categorized into two groups: those with a low QoL and those with a high QoL. In the univariate analyses of the composite QoL score concerning demographic and clinical determinants, both receiving psychological support (*p* = 0.001) and reporting side effects to the physician (*p* = 0.001) were identified as significant. Multivariate analyses further confirmed that receiving psychological support and reporting side effects to the physician significantly impacted QoL. Quality of life was lower among individuals who received psychological support (OR: 0.36, 95% CI: 0.19–0.67, *p* = 0.002). The composite QoL score was notably lower in those who reported side effects to their physicians (OR: 0.28, 95% CI: 0.13–0.64, *p* = 0.001) (Table 3).

## 3. Discussion

In this study, the QoL and symptom burden associated with the side effects of endocrine therapy were thoroughly assessed in patients with early-stage breast cancer undergoing adjuvant endocrine therapy. This assessment utilized composite scores derived from question sets inspired by the conceptual framework of established measurement tools. The internal consistency of the composite symptom burden score was deemed acceptable, with a Cronbach’s alpha coefficient of 0.717. Multivariate analysis revealed that a history of chemotherapy, use of ovarian suppression, and reporting of side effects to a physician were independently associated with a higher symptom burden. Conversely, age, menopausal status, and class of endocrine therapy administered were not independent predictors of the composite symptom burden in the multivariate model. In a separate model, a lower composite quality of life (QoL) score was significantly associated with having received psychological support and reporting side effects to a physician. This pattern suggests that the experience of side effects and the behavior of seeking and reporting healthcare are often concurrent and mutually reinforcing processes within the same individual. In clinical practice, patients experiencing more severe symptoms are naturally more inclined to seek assistance.

The management of hormone receptor-positive (HR+) early-stage breast cancer has been revolutionized by the widespread use of adjuvant endocrine therapy (AET), a class of treatment that has unequivocally improved recurrence-free and overall survival outcomes [8]. However, the long-term nature of AET, typically prescribed for five to ten years, imposes a significant and often underestimated burden of side effects that can profoundly impair quality of life (QoL) and lead to premature discontinuation of this life-saving treatment [9]. This report provides a comprehensive analysis of the symptom burden and QoL associated with AET, integrating findings from a cross-sectional study with an extensive review of contemporary evidence to propose a new patient-centered standard of care.

The European Organization for Research and Treatment of Cancer has developed tools that offer an established framework for patient-centered measurements. Aaronson et al. [10] and Sprangers et al. [11] have demonstrated the reliability and validity of the 30-item core QoL questionnaire and the 23-item breast cancer-specific module across various settings. The item architecture of these instruments systematically captures essential symptom domains, including fatigue, pain, gastrointestinal complaints, and physical, emotional, and social functioning. In our study, the construction of the question pool, informed by the conceptual domains of this architecture, provided a methodological foundation for the content validity of the composite scores. This approach, aligned with the developmental trajectories of Aaronson et al. and Sprangers et al., reinforces the robustness of our findings in the context of the measurement theory.

The methodological validity of these findings is anchored in their alignment with internationally recognized standards for patient-reported outcome (PRO) measurements. The study’s questionnaire design was informed by the conceptual architecture of the European Organization for Research and Treatment of Cancer (EORTC) Quality of Life Questionnaires, specifically the QLQ-C30 core instrument and its breast cancer-specific module (BR23). The enduring relevance and validity of these EORTC tools are continuously reaffirmed by their central role in contemporary clinical trials and large-scale cohort studies, where they serve as the gold standard for capturing the multidimensional impact of cancer therapies on patient functioning and well-being [12,13].

The impact of adjuvant endocrine therapy strategies on QoL and symptom profiles in premenopausal women has been extensively documented in several randomized trials using patient-reported outcomes. Bernhard et al. [14] observed that symptoms related to hormone deprivation, such as vaginal dryness and sexual dysfunction, were more pronounced in the group receiving exemestane combined with ovarian suppression compared to the group receiving tamoxifen with ovarian suppression. However, differences in overall QoL were minimal and evolved over time. Similarly, Ribi et al. [15] demonstrated that the addition of ovarian suppression to tamoxifen increased the incidence of vasomotor and other symptoms but did not result in persistent and significant differences in overall QoL. Our study’s finding that ovarian suppression independently predicts a higher symptom burden aligns with the findings of Bernhard et al. and Ribi et al., suggesting that the symptom experience in premenopausal women is influenced more by the “degree of suppression of the endocrine environment” rather than the dichotomy between “tamoxifen or aromatase inhibition.”

In a postmenopausal context, Fallowfield et al. [16] evaluated patient-reported outcomes in an adjuvant study involving Arimidex and Tamoxifen, either alone or in combination. Their findings indicated that endocrine symptom patterns varied between treatment arms within the first two years, with the anastrozole arm experiencing more vaginal dryness and decreased sexual desire. However, no clinically significant differences were observed in the overall and outcome-related QoL measures. This observation aligns with our multivariate analysis, which demonstrated that the class of endocrine treatment did not remain an independent predictor of the composite symptom burden when other factors were controlled. Thus, although specific symptom clusters associated with each agent exist, their overall impact on the patient experience may be limited when considering the broader clinical context.

The association between a history of chemotherapy and increased symptom burden is substantiated by the patient-reported literature. Ribi et al. demonstrated that the residual effects of chemotherapy, including fatigue, paresthesia, cognitive complaints, and musculoskeletal pain, can exacerbate the overall symptom burden during the endocrine therapy phase. Consequently, our findings align with those of Ribi et al., indicating that the cumulative toxicity of multi-step treatment persists during the endocrine treatment phase.

The association between reporting side effects to physicians and both increased symptom burden and reduced QoL should be interpreted cautiously. Dueck et al. [7] demonstrated that the Common Terminology Criteria for Adverse Events Patient Reporting Version validly and reliably measures symptom frequency, severity, and impact on daily life. Basch et al. [17] and Di Maio et al. [18] identified systematic discrepancies between clinician assessments and patient reports, with clinicians often reporting lower severity, particularly for subjective toxicity. The programmatic framework of the National Cancer Institute’s Patient-Reported Outcomes [19] also underscores the complementary nature of patient-reported data. This body of evidence suggests that the likelihood of reporting and seeking healthcare increases with the severity of symptoms. Therefore, the association of the “reporting side effects to the physician” variable with poorer scores in our study aligns with the directionality demonstrated by Dueck et al., Basch et al., and Di Maio et al.: reporting itself is not the cause of deterioration, but rather an indicator of symptom severity.

The concurrent occurrence of receiving psychological support and experiencing a lower QoL should be understood through the lens of selection dynamics, rather than as an indication of the ineffectiveness of psychosocial interventions. Faller et al. [20] demonstrated through a systematic review and meta-analysis of randomized controlled trials that psycho-oncological interventions yield small to moderate beneficial effects in alleviating emotional distress and enhancing QoL. The relationship observed in the cross-sectional context likely reflects the increased propensity of individuals with elevated distress levels to seek support and be referred for help. Consequently, this does not contradict the findings of Faller et al., but rather offers a complementary perspective within the different contexts of timing, design, and patient selection.

A multitude of studies in the field consistently identify a cluster of symptoms, including but not limited to fatigue, sleep disturbance, anxiety, depression, and cognitive dysfunction [21,22]. The finding in this study that “receiving psychological support” was associated with a lower QoL strongly suggests that this cluster is quite prevalent in the study population. The classic triad of hot flashes, night sweats, and cold sweats is indicative of estrogen deficiency and is frequently observed in patients receiving AET [23]. A further common affliction that significantly impacts patients’ QoL and treatment adherence is a cluster of arthralgia and myalgia, particularly in cases associated with aromatase inhibitors [24].

Our analysis of single-variable observations related to complementary and alternative approaches revealed that patient behaviors were closely associated with their symptom experiences. Dehghan Nayeri et al. [25] conducted a systematic review of complementary and alternative approaches in breast cancer, indicating that certain mind–body interventions may improve specific aspects of quality of life. However, the evidence for herbal products remains heterogeneous and methodologically limited. Yen H-R et al. [26] examined the potential interactions between tamoxifen and commonly used natural products and medications, warning that such interactions could modify the pharmacokinetics or pharmacodynamics of endocrine therapy. The observation of an increased symptom burden among patients who resorted to complementary and alternative approaches in our study aligns with a behavioral pattern, suggesting that individuals with more pronounced symptoms seek relief through these methods. This finding emphasizes the safety and interaction concerns highlighted by Yen et al. from a clinical management perspective. Furthermore, the Oncology Nursing Society’s guidance on complementary and integrative approaches in supportive care [27] advocates regular inquiries about the use of complementary products and careful management of potential interactions. Our data, by identifying a symptom-defined subgroup, reinforce this recommendation.

The existing literature on the practical implications of incorporating patient-reported outcomes into routine care through digital tools indicates that such integration can facilitate early symptom detection, expedite access to supportive interventions, and enhance clinical outcomes in specific contexts. Basch et al. [28] demonstrated in a randomized trial that real-time symptom monitoring based on patient reporting within routine oncology care can improve not only patient-reported outcomes but also overall survival rates. These findings are consistent with our practical conclusions, suggesting that proactive, algorithm-driven supportive care should be informed by systematic patient reporting, particularly in subgroups with a high symptom burden, such as those with a history of chemotherapy and those undergoing OFS.

From a clinical practice perspective, we propose four complementary strategies. First, patients must receive structured information regarding treatment-specific symptom profiles and concrete management strategies before commencing AET. Second, during follow-up, healthcare teams should systematically collect patient-reported data at regular intervals and promptly implement evidence-based supportive measures tailored to reported symptom domains. Third, the use of complementary and alternative products should be systematically inquired about at each visit, and potential drug-product interactions should be managed in collaboration with the oncology pharmacy unit. This recommendation aligns with the warnings of Yen et al. and the guidance provided by the Oncology Nursing Society. Fourth, access to evidence-based psycho-oncological interventions should be facilitated for patients with significant psychological needs. It is important to note that our cross-sectional relationship does not contradict the beneficial effects demonstrated by Faller et al., but rather reflects the initial level of distress.

While the findings of this study from a Turkish cohort are valuable, it is important to acknowledge the influence of cultural and racial contexts on symptom perception and reporting behaviors. Comprehensive studies from the US have demonstrated that Black women experience a higher symptom burden, lower adherence to adjuvant endocrine therapy (AET), and a more significant patient-physician communication gap than White women. This communication gap is a significant driver of care disparities [29,30]. Conversely, studies that compare Dutch and Japanese patients demonstrate substantial disparities in their perceptions of the disease, the mechanisms they employ to cope, and the impact of symptoms on their quality of life [31]. These cultural frameworks influence how symptoms are perceived and communicated. This underscores the necessity to circumvent the oversimplification of the study’s findings and to contextualize them as a culturally specific data point. This underscores the pressing need for international, cross-cultural research to elucidate the variability in symptom-reporting behaviors and their relationship with quality of life across diverse populations. The efficacy of interventions is contingent upon their adaptation to the unique communication styles, illness perceptions, and systemic barriers of diverse patient populations.

The limitations of our study include its single-center and cross-sectional design, the expression of composite scores based on the global burden rather than subscales, the use of median-based thresholds that may not perfectly align with clinical decision boundaries, and the lack of external validation of the results. Additionally, while the chi-square test was used for initial variable screening, employing univariate logistic regression could have offered further details on the strength of individual associations. Furthermore, the small sample sizes for certain subgroups, particularly patients who sought support from non-physician health professionals (n = 21) or used complementary and alternative medicines (n = 18), limit the statistical robustness of the findings related to these variables and may affect their generalizability. Nevertheless, the underlying rationale for our decision to exclude these two groups from the analysis is that they embody the originality of our study and the primary point we wish to emphasize. The primary objective of our study was to draw attention to the existence and tendencies of these patient groups, which are often overlooked or underrepresented in the literature. These groups underscore a substantial gap in current clinical practice and introduce a unique awareness to our study, distinguishing it from other studies. Consequently, despite the constrained statistical power, it is hypothesized that the presentation of data on these groups will function as a foundational starting point and a source of hypotheses for the development of more comprehensive research in the future.

The association between reporting side effects to physicians and both increased symptom burden and reduced QoL should be interpreted cautiously. This relationship is not one of causation; rather, our findings are consistent with evidence suggesting that the likelihood of reporting and seeking healthcare increases with the severity of symptoms. Therefore, reporting behavior in our study should be seen as an indicator of a higher symptom burden, not its cause. Symptom burden and quality of life are affected by clinical history, behavioral dynamics (which are associated with symptom severity, such as reporting to a physician), and supportive factors.

Conversely, the strengths of our study include the content design of our tools being based on the European Organization for Research and Treatment of Cancer scales and the Common Terminology Criteria for Adverse Events Patient Reporting Version frameworks, and the internal consistency coefficient being at an acceptable level. Future research should investigate the effects of prospective and longitudinal designs that elucidate symptom trajectories over time and patient-reported early intervention programs on treatment continuity and clinical outcomes. It is also recommended to evaluate the measurement capabilities of the updated version of the breast cancer-specific module [32].

## 4. Conclusions

Symptom burden and quality of life in patients with HR + breast cancer undergoing adjuvant hormone therapy are affected by clinical history (such as chemotherapy and OFS), behavioral dynamics (such as reporting to the physician), and supportive factors (including the use of TAT and psychological support). These findings highlight the critical importance of improving patient-physician communication, implementing PRO-based systematic monitoring, and developing personalized and supportive care strategies.

## Figures and Tables

**Table 1:** Demographic and Clinical Characteristics of the Patient Population Participating in the Survey.

	n = 191
Age (Med.)	54 (46–61)
Gender	
Female	191 (100%)
Menopausal Status	
Pre-Perimenopause	73 (38.2%)
Postmenopausal	118 (61.8%)
ECOG PS	
0–1	191 (100%)
Comorbid Disease	
None	60 (31.4%)
Yes	131 (68.6%)
Diagnosis Stage	
1	74 (38.7%)
2	72 (37.7%)
3	45 (23.6%)
C-Erb B2	
Negative	146 (76.4%)
Positive	45 (23.6%)
Chemotherapy History	
None	33 (17.3%)
Yes	156 (81.7%)
Unknown	2 (1.0%)
History of Radiotherapy	
None	26 (13.6%)
Yes	165 (86.4%)
Type of Hormone Therapy	
Tamoxifen	71 (37.2%)
Aromatase Inhibitor	120 (62.8%)
Using OFS	
None	135 (70.7%)
Yes	56 (29.3%)
Educational Status	
Elementary-Middle School	95 (49.7%)
High School-Undergraduate-Graduate	90 (47.1%)
Unknown	6 (3.1%)
Marital Status	
Married	131 (68.6%)
Single/Widowed	56 (29.3%)
Unknown	4 (2.1%)
Employment Status	
Employed	43 (22.5%)
Not actively working	148 (77.5%)

Med: Median ECOG PS: Eastern Cooperative Oncology Study Group Performance Score OFS: Ovarian Function Suppression.

**Table 2:** Clinical and Demographic Determinants of the Composite Symptom Burden Score.

	Univariate Analyses		Multivariate Analysis	
	Composite Symptom Burden Score (Low/High)	*p* Value	Odds Ratio (95% Confidence Interval)	*p* Value
Age		0.001		0.240
≤50 (Ref.)	31.4%/68.6%			
>50	56.2%/43.8%		0.54 (0.19–1.51)	
Menopausal Status		0.005		0.759
Postmenopausal (Ref.)	55.1%/44.9%			
Pre-Perimenopause	34.2%/65.8		1.27 (0.28–5.77)	
Stage		0.189		
1 (Ref.)	55.4%/44.6			
2	41.7%/58.3%		1.24 (0.53–2.92)	0.619
3	42.2%/57.8%		1.01 (0.38–2.70)	0.981
Comorbidity		0.074		0.889
None (Ref.)	42.7%/57.3			
Yes	56.7%/43.3		0.94 (0.42–2.13)	
C-ErbB2		0.786		
Negative (Ref.)	46.6%/53.4%			
Positive	48.9%/51.1			
Chemotherapy History		0.013		0.006
None (Ref.)	66.7%/33.3			
Yes	42.9%/57.1%		3.75 (1.46–9.69)	
History of Radiotherapy		0.597		
None (Ref.)	42.3%/57.7			
Yes	47.9%/52.1			
Type of Hormone Therapy		0.025		0.290
Tamoxifen (Ref.)	36.6%/63.4			
Aromatase Inhibitor	53.3%/56.7		1.85 (0.59–5.77)	
OFS Use		0.002		0.003
None (Ref.)	54.4%/45.6%			
Yes	29.1%/70.9%		3.29 (1.51–7.15)	
Educational Status		0.493		
Primary-Secondary School	49.5%/50.5			
High School-Undergraduate-Graduate	44.4%/55.6			
Marital Status		0.243		0.108
Married (Ref.)	44.3%/55.7			
Single/Widowed	53.6%/46.4		0.55 (0.26–1.14)	
Employment Status		0.661		
Employed (Ref.)	44.2%/45.8			
Not actively working	48.0%/52.0			
Receiving Psychological Support		0.022		0.087
None (Ref.)	51.3%/48.7%			
Yes	30.8%/69.2%		2.09 (0.90–4.87)	
Visiting a Non-Physician Healthcare Professional		0.609		
No (Ref.)	46.5%/53.5			
Yes	52.4%/47.6			
Report Side Effects to Your Doctor		<0.001		<0.001
No (Ref.)	66.3%/33.7			
Yes	30.8%/69.2%		3.52 (1.80–6.88)	
Herbal Product/Alternative Medicine Application		0.007		0.011
No (Ref.)	50.3%/49.7			
Yes	16.7%/83.3%		7.27 (1.57–33.63)	

Ref: Reference OFS: ovarian function suppression.

**Table 3:** Clinical and Demographic Determinants of the Composite Quality of Life Score.

	Univariate Analyses		Multivariate Analysis	
	Composite Symptom Burden Score (Low/High)	*p* Value	Odds Ratio (95% Confidence Interval)	*p* Value
Age		0.07		0.227
≤50 (Ref.)	52.9%/47.1%			
>50	38.8%/61.2%		1.52 (0.77–2.97)	
Menopausal Status		0.177		0.690
Postmenopausal (Ref.)	39.8%/60.2%			
Pre-Perimenopause	50.7%/49.3%		0.81 (0.29–2.25)	
Stage		0.334		
1 (Ref.)	37.8%/62.2%			
2	50.0%/50.0%		0.53 (0.25–1.10)	0.086
3	44.4%/55.6%		0.63 (0.28–1.44)	0.272
Comorbidity		0.876		
None (Ref.)	43.5%/56.5			
Yes	45.0%/55.0			
C-ErbB2		0.732		
Negative (Ref.)	43.2%/56.8			
Positive	46.7%/53.3			
Chemotherapy History		0.816		
None (Ref.)	42.4%/57.6			
Yes	44.2%/55.8			
History of Radiotherapy		0.853		
No (Ref.)	42.3%/57.7			
Yes	44.2%/55.8			
Type of Hormone Therapy		0.815		
Tamoxifen (Ref.)	45.1%/54.9			
Aromatase Inhibitor	43.3%/56.7			
OFS Use		0.630		
None (Ref.)	42.6%/57.4			
Yes	47.3%/52.7			
Education		0.378		0.630
Primary-Secondary School	41.1%/58.9			
High School-Undergraduate-Graduate	47.8%/52.2		1.19 (0.59–2.40)	
Marital Status		0.107		0.176
Married (Ref.)	39.7%/60.1			
Single/Widowed	53.6%/46.4		0.62 (0.31–1.24)	
Employment Status		0.601		
Employed (Ref.)	39.5%/60.5			
Not actively working	45.3%/54.7%			
Receiving Psychological Support		0.001		0.002
None (Ref.)	38.0%/62.0			
Yes	69.2%/30.8%		0.36 (0.19–0.67)	
Visiting a Non-Physician Healthcare Professional		0.817		
No (Ref.)	43.5%/56.5			
Yes	47.6%/52.4			
Reporting Side Effects to Doctor		0.001		0.001
No (Ref.)	30.2%/69.8%			
Yes	54.8%/45.2%		0.28 (0.13–0.64)	
Herbal Product/Alternative Medicine Application		0.327		0.181
No (Ref.)	42.8%/57.2			
Yes	55.6%/44.4%		0.47 (0.16–1.42)	

## Data Availability

The data presented in this study are available on request from the corresponding author. The data are not publicly available due to privacy and ethical restrictions as they contain information that could compromise the privacy of research participants.
